# Suspected COVID-19 Immunization-Induced Probable Catastrophic Antiphospholipid Syndrome

**DOI:** 10.7759/cureus.27313

**Published:** 2022-07-26

**Authors:** Elizabeth A Seeley, Markie Zimmer, Ramona Berghea

**Affiliations:** 1 Internal Medicine, Beaumont Health, Royal Oak, USA

**Keywords:** systemic lupus erythromatosus, internal medicine and rheumatology, immune-hematology, heart failure with reduced ejection fraction, covid-19 vaccine, catastrophic antiphospholipid syndrome (caps)

## Abstract

In this report, we describe the case of a woman with suspected COVID-19 immunization-induced probable catastrophic antiphospholipid syndrome. The patient is a 35-year-old female with a past medical history significant for antiphospholipid syndrome, not on anticoagulation, who presented with a 5-day history of abdominal pain and distention, nausea, vomiting, and shortness of breath. She had received her first dose of the Pfizer COVID-19 vaccine one day prior to the onset of symptoms. After extensive workup at an outside hospital, she was found to be in acute heart failure exacerbated by severe mitral and tricuspid regurgitation. She was transferred to our hospital for escalation of care. EKG showed evidence of prior inferior and septal myocardial infarction. Transesophageal echocardiogram (TEE) showed reduced ejection fraction, severe mitral and tricuspid regurgitation, and a left ventricular thrombus. Cardiac MRI showed subendocardial late gadolinium enhancement indicative of ischemia. However, CTA of the coronary vessels showed no signs of obstruction. Therefore, her acute heart failure was thought to be due to small vessel thrombosis secondary to antiphospholipid syndrome. During admission, she had several absence seizure-like episodes. CT head showed several hypodensities of the deep white matter and brain MRI demonstrated multiple hyperintense T2 FLAIR signal foci with restriction diffusion and enhancement involving the cerebral hemisphere, consistent with subacute strokes attributed to being secondary to antiphospholipid syndrome or embolic from the left ventricular thrombus. She was treated with heparin for suspected catastrophic antiphospholipid syndrome and high-dose corticosteroid therapy for concomitant Systemic Lupus Erythematosus (SLE). She was discharged in a stable condition.

## Introduction

Antiphospholipid syndrome is characterized by vascular thrombosis and/or pregnancy morbidity. The diagnostic criteria for antiphospholipid syndrome were originally described by the Sapporo preliminary classification criteria for antiphospholipid syndrome and have since been revised [1]. Deep vein thrombosis is the most common venous manifestation, while stroke is the most common arterial manifestation. Here, we report a case of likely multi-organ microthrombosis secondary to antiphospholipid syndrome.

## Case presentation

Signs and symptoms

A 35-year-old female presented to an outside hospital with a 5-day history of abdominal pain and distention, nausea, vomiting, and shortness of breath. The patient’s past medical history was significant for antiphospholipid syndrome, which had been diagnosed 7 months prior in an evaluation of elevated activated partial thromboplastin time (aPTT) found in pre-operative labs. Although we were unable to access the lab results from her hematologist, the patient reported undergoing testing that had confirmed the diagnosis of antiphospholipid syndrome. She also endorsed two prior miscarriages, one at 9 weeks of gestation and one at 13 weeks of gestation.

After initial evaluation at the outside hospital, the patient was found to have heart failure with reduced ejection fraction (HFrEF) and severe mitral and tricuspid regurgitation. She was transferred to our hospital for evaluation for structural heart disease. On admission to our hospital, the patient was afebrile (36.7°C) with a blood pressure of 107/63 mmHg and a heart rate of 78 beats per minute. On review of systems, the patient endorsed swelling of her hands and feet, face rash, abdominal pain, nausea, vomiting, cough, joint pain, myalgias, fatigue, and a 20-30lb weight gain, as well as confusion and memory loss. Physical exam revealed an anxious female with a facial rash consistent with acne, Grade II/VI holosystolic murmur at the apex, crackles in bilateral lung bases, and 1+ pitting edema of the lower extremities bilaterally. There were no signs of ischemia or cyanosis of the extremities.

Labs and imaging

Initial lab results showed leukocytosis, normocytic anemia, thrombocytopenia, acute kidney injury and elevated liver enzymes, and signs of cardiac myocyte injury and strain (Table 1). Urinalysis showed hematuria and proteinuria without casts (Table 2). The autoimmune workup confirmed the patient had triple-positive antiphospholipid syndrome (Table 3).

**Table 1:** Lab investigations performed. WBC: white blood count, RBC: red blood count, MCV: mean corpuscular volume, MCH: mean corpuscular hemoglobin, MCHC: mean corpuscular hemoglobin concentration, RDW-CV: red cell distribution width, GFR: glomerular filtration rate, INR: international normalized ratio, aPTT: activated partial thromboplastin time

Component	Ref Range & Units	Value
WBC	3.3 - 10.7 bil/L	14.1
RBC	3.87 - 5.08 tril/L	3.27
Hemoglobin	12.1 - 15.0 g/dL	8.9
Hematocrit	35.4 - 44.2 %	31.2
MCV	80 - 100 fL	95
MCH	28 - 33 pg	27
MCHC	32 - 35 g/dL	29
RDW-CV	12 - 15 %	20
Platelets	150 - 400 bil/L	82
Nucleated Red Blood Cells	<=0.0 %	0.2
Sodium	135 - 145 mmol/L	129
Potassium	3.5 - 5.2 mmol/L	5.9
Chloride	98 - 111 mmol/L	94
Carbon Dioxide (CO2)	20 - 29 mmol/L	16
Anion Gap	5 - 17	19
Glucose	60 - 99 mg/dL	111
Blood Urea Nitrogen (BUN)	7 - 25 mg/dL	44
Creatinine	0.50 - 1.10 mg/dL	2.15
GFR Non African American	>=60 mL/min/1.73m2	29
Calcium	8.5 - 10.5 mg/dL	8.9
Protein Total	6.4 - 8.3 g/dL	5.9
Albumin	3.5 - 5.1 g/dL	2.8
Globulin	2.2 - 4.0 g/dL	3.1
Albumin/Globulin Ratio		0.9
Alkaline Phosphatase	33 - 120 U/L	196
Aspartate Aminotransferase (AST)	<35 U/L	315
Alanine Aminotransferase (ALT)	8 - 37 U/L	299
Bilirubin Total	0.3 - 1.2 mg/dL	1.0
Prothrombin Time	9.2 - 13.5 Seconds	21.6
INR		1.9
aPTT	25.0-38.0 Seconds	55.9
Troponin I	<=0.03 ng/mL	0.29
Comment: Normal: <0.04		
Indeterminate: 0.04-0.29		
Suggestive of Myocardial Damage: >=0.30		
B Type Natriuretic Peptide (BNP)	0 - 100 pg/mL	2,646

**Table 2:** Urinalysis upon admission. RBC: red blood count, WBC: white blood count

Component	Ref Range & Units	Value
Color		Yellow
Clarity	Clear	Clear
Glucose	Negative (mg/dL)	50
Bilirubin	Negative	Negative
Ketones	Negative (mg/dL)	Negative
Specific Gravity, Urine	1.005 - 1.030	1.012
Blood	Negative	1+
pH	5.0 - 8.0	6.0
Urine Protein:Creatinine Ratio	0.00-0.20	1.03
Urobilinogen	<2.0 mg/dL	<2.0
Nitrites	Negative	Negative
Leukocyte Esterase	Negative	Negative
RBC	0-2 (Negative) /hpf	0-2 (Negative)
WBC	0-5 (Negative) /hpf	0-5 (Negative)
Epithelial, Squamous	Negative /lpf	1-5
Casts, Hyaline	0-2 (Negative) /lpf	3-5
Bacteria	Negative /hpf	1+

**Table 3:** Autoimmune investigation results. ANA: antinuclear antibody, DRVVT: Diluted Russel Viper Venom Time

Component	Ref Range & Units	Value
ANA Titer & Pattern	< 1:80	1:320, homogeneous pattern
Double Stranded DNA Antibody, IgG	<100.0 AU/mL	25.0
Complement, C3	82-193 mg/dL	55
Complement, C4	10-43 mg/dL	8
B2 Glycoprotein IgG Antibody	<= 20 SGU	21
Cardiolipin Antibody IgG	< 15 GPL	72.0
Cardiolipin Antibody IgM	< 12.5 MPL	21.7
DRVVT	27-45 sec	121
Lupus Anticoagulant	30.3-43.2 sec	100.0

**Figure 1:**
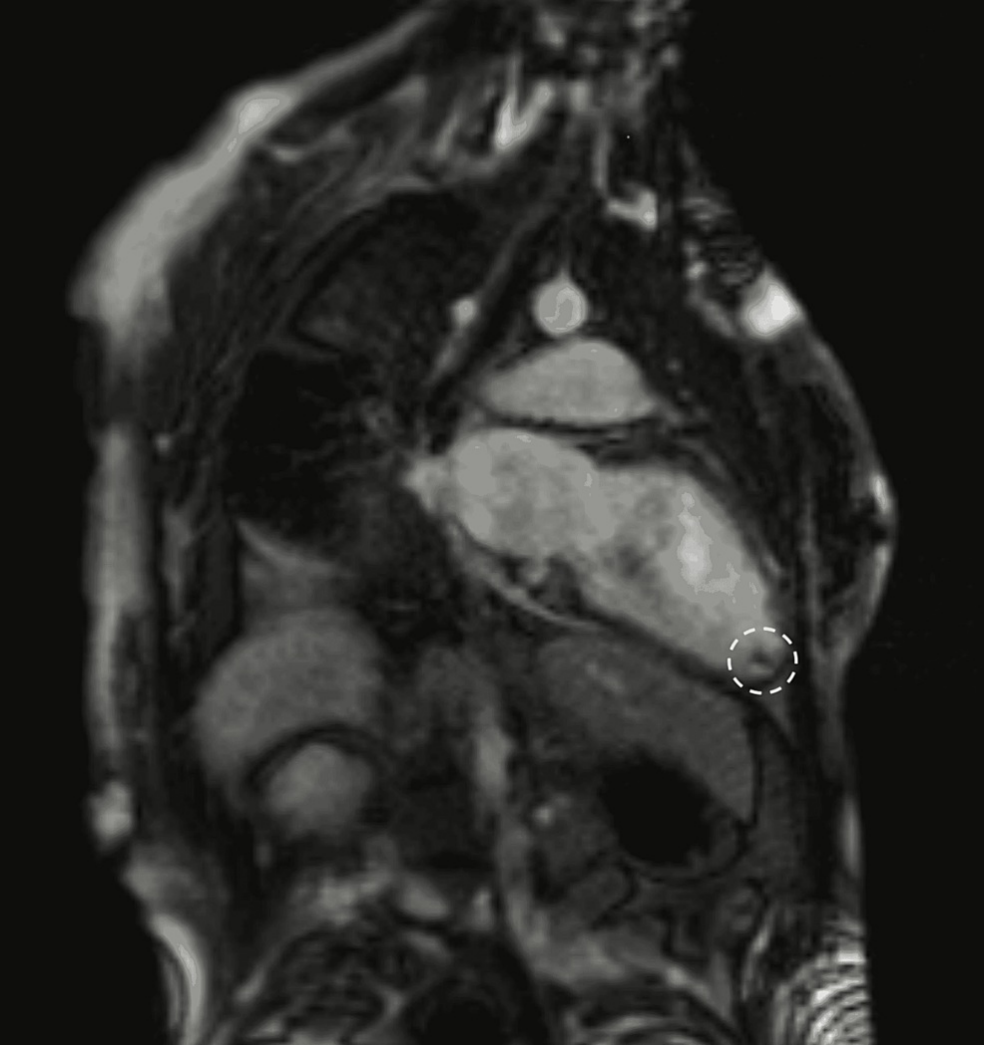
Cardiac MRI showing apical left ventricular thrombus (white circle).

Electrocardiogram (EKG) showed evidence of a prior inferior and septal myocardial infarction. Transesophageal echocardiogram (TEE) showed an estimated left ventricular ejection fraction (LVEF) of 47%, estimated pulmonary artery pressure of 32mmHg, thickened posterior mitral valve leaflet, and severe mitral and tricuspid regurgitation. CT angiography of the chest showed no evidence of a large central pulmonary embolism, but did show a large right-sided pleural effusion and a small left-sided pleural effusion. Thoracentesis drained 1800mL of transudative fluid; cytology was negative for malignancy and there were no organisms identified on the culture. Cardiac MRI showed an LVEF of 35%, subendocardial late gadolinium enhancement of the basal and mid-inferolateral wall, mid-anteroseptal wall, and the septal, inferior, and lateral walls of the apex involving approximately 50% of the myocardial thickness, apex and basolateral akinesis, severe mitral and tricuspid regurgitation, and a mobile left ventricular thrombus (1cm x 1cm) (Figure 1). CT angiography of the coronary arteries showed no evidence of obstructive coronary artery disease.

**Figure 2:**
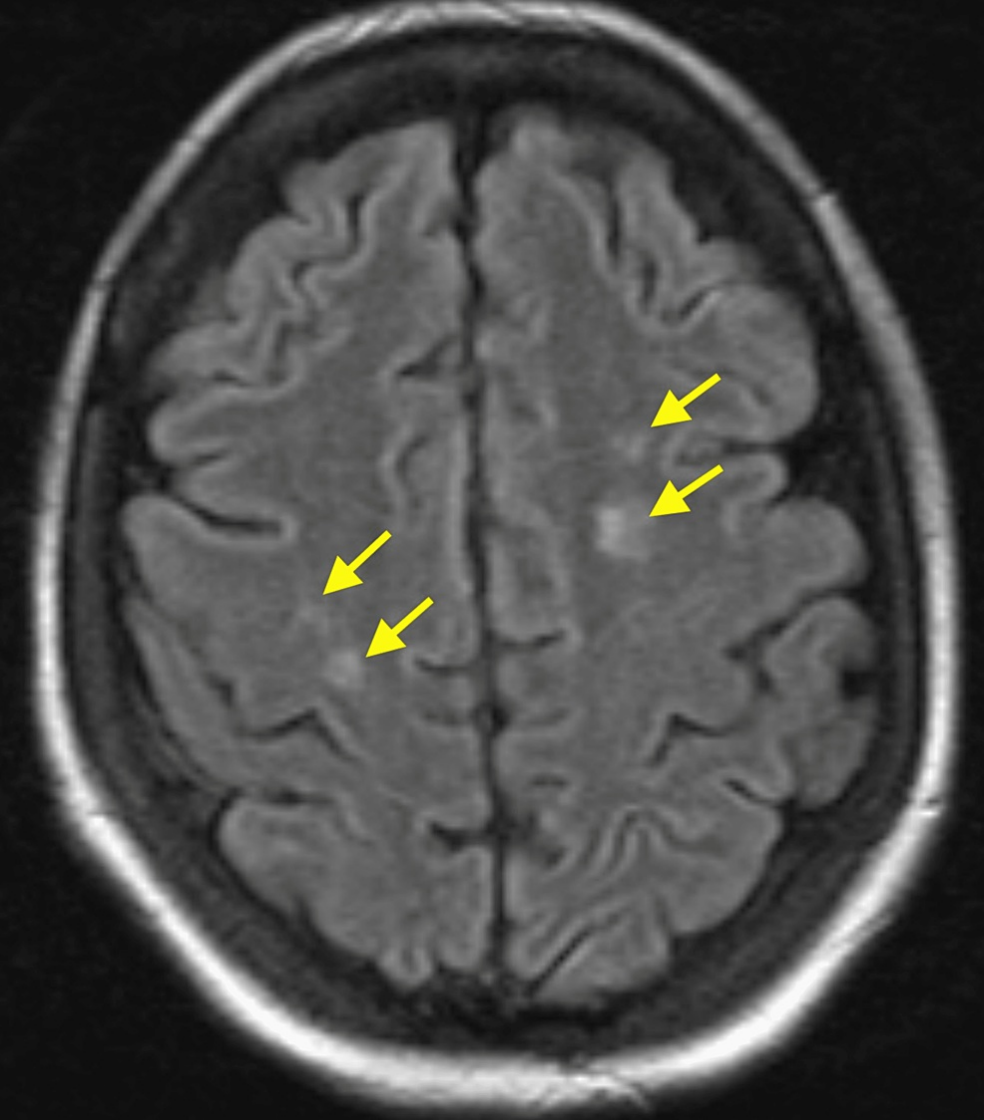
FLAIR sequence on brain MRI depicting multiple hyperintense signal foci (yellow arrows).

In regards to her confusion and memory loss, non-contrast CT head showed hypodensities of the deep white matter of both frontal lobes and the left parietal lobe. The patient also had several seizure-like episodes throughout the admission. Short-term and continuous electroencephalogram (EEG) showed no epileptiform activity and repeat head CTs were stable. Brain MRI demonstrated multiple hyperintense T2 FLAIR signal foci with restriction diffusion and enhancement involving the cerebral hemisphere (Figure 2). The patient then underwent a lumbar puncture. Cerebrospinal fluid analysis showed normal protein (40 mg/dL) but elevated glucose (110 mg/dL), and was negative for infectious causes of meningitis and encephalitis (Table 4). CTA of the head and neck showed no evidence of large vessel arterial occlusion.

**Table 4:** Meningitis and encephalitis panel results.

Component	Ref Range & Units	Result
Escherichia coli K1	Not Detected	Not Detected
Haemophilus influenza	Not Detected	Not Detected
Listeria monocytogenes	Not Detected	Not Detected
Neisseria meningitidis	Not Detected	Not Detected
Streptococcus agalactiae	Not Detected	Not Detected
Streptococcus pneumoniae	Not Detected	Not Detected
Cryptococcus neoformans/gattii	Not Detected	Not Detected
Cytomegalovirus	Not Detected	Not Detected
Human Herpes Virus 6	Not Detected	Not Detected
Human Parechovirus	Not Detected	Not Detected
Varicella Zoster Virus	Not Detected	Not Detected
Enterovirus	Not Detected	Not Detected
Herpes Simplex Virus 1	Not Detected	Not Detected
Herpes Simplex Virus 2	Not Detected	Not Detected

Differential Diagnosis

The differential diagnosis for acute HFrEF with valvular disease is broad. Infective endocarditis was considered primarily due to signs of glomerulonephritis, including hematuria on urinalysis. The patient had no history of intravenous drug use, rheumatic heart disease, or any other preexisting valve disease. She underwent transesophageal and transthoracic echocardiograms, neither of which indicated the presence of any vegetations on the cardiac valves. Additionally, the patient remained afebrile, and repeat blood cultures were negative throughout admission.

Autoimmune myocarditis was also considered. The patient had a positive ANA titer at 1:320, hypocomplementemia, proteinuria, and a history of joint arthralgias which indicated a diagnosis of SLE based on the 2019 European Alliance of Associations for Rheumatology/American College of Rheumatology classification criteria. Cardiac manifestations of SLE include valvular vegetations and myocarditis, both of which can contribute to the development of heart failure in these patients. Echocardiograms showed no vegetations.

Ischemic cardiomyopathy was another consideration, as the EKG showed evidence of prior inferior and septal myocardial infarction, and troponin was elevated. This patient’s primary risk factor for coronary artery disease was a 5-pack-year smoking history, though she had quit one year prior to presentation. CT angiography of the coronary arteries showed no obstructive coronary artery disease.

Post-vaccine myocarditis was also a diagnosis under consideration, as this patient had received the first dose of the Pfizer mRNA COVID-19 vaccine one day prior to the onset of symptoms. There is a known risk of the development of myocarditis after receiving the COVID-19 mRNA vaccines [2-4]. These cases have typically been seen in young adult men presenting with chest pain onset within a few days of vaccination and have rarely resulted in acute heart failure [3,4].

Notably, the patient also had a confirmed diagnosis of antiphospholipid syndrome. The most common cardiac manifestation of antiphospholipid syndrome is valvulopathy, including valve thickening and valve vegetations, which can rarely cause clinically significant valve regurgitation [5-8]. TTE performed one year prior was unremarkable but at the time of this admission, showed thickening of the mitral valve and severe mitral regurgitation. Antiphospholipid syndrome has also been associated with myocardial ischemia. Accelerated development of atherosclerosis and coronary thromboembolism has been known to cause myocardial infarction in these patients. However, antiphospholipid syndrome has also been found to cause myocardial dysfunction due to microvascular thrombosis and widespread myocardial ischemia and necrosis [5,8].

The subendocardial distribution of late gadolinium enhancement seen on cardiac MRI was indicative of ischemic etiology, rather than inflammatory or vaccine-related myocarditis, as these are generally associated with late gadolinium enhancement in an epicardial and mid-wall or subepicardial distribution, respectively [9,10,11]. CT angiography was then performed to evaluate the coronary arteries, which interestingly showed no evidence of coronary artery disease or obstruction. The final diagnosis was therefore myocardial infarction without obstructive coronary arteries secondary to small vessel thrombosis in the setting of antiphospholipid syndrome.

Catastrophic Antiphospholipid Syndrome (CAPS) is a form of antiphospholipid syndrome associated with multi-organ small vessel thrombosis. CAPS diagnosis requires 1) evidence of involvement of three or more organs, systems, and/or tissues; 2) development of symptoms within one week; 3) confirmation of small vessel thrombosis by histopathology in at least one organ or tissue; 4) laboratory confirmation of the presence of antiphospholipid antibodies [12]. CAPS is generally treated with anticoagulation, pulse-dose corticosteroids followed by a taper, and plasmapheresis and/or intravenous immunoglobulin [13]. Although there was evidence of multi-organ involvement (likely of ischemic origin) including the central nervous system and heart, there was not enough evidence to diagnose this patient with CAPS.

Another area of focus was identifying the cause of the hypodensities seen on the head CT. Brain MRI showed hyperintense T2 FLAIR signal foci with restriction diffusion and enhancement. These were thought to be consistent with an inflammatory process, demyelination, or subacute stroke with luxury perfusion, which is caused by increased blood flow to an area of recently infarcted brain tissue. Benign CSF on lumbar puncture argued against infectious etiology and active inflammatory central nervous system disease secondary to autoimmune disease. Therefore, the primary diagnosis was small vessel strokes with luxury perfusion. The small vessel strokes may have been secondary to small vessel thrombosis in the setting of antiphospholipid syndrome or may have been cardioembolic from the left ventricular thrombus.

The patient also had several episodes of absence seizure-like activity during admission. Interestingly, antiphospholipid antibodies may also play a part in the pathogenesis of seizure development via thrombotic mechanisms or direct depolarization of neurons [14]. We were unable to document epileptiform activity on repeat EEGs, but seizure activity may also be associated with antiphospholipid syndrome.

It should be noted that the vectorial COVID-19 vaccines have been associated with vaccine-induced thrombotic thrombocytopenia (VITT) [15]. The most common presentation of VITT is cerebral venous sinus thrombosis, although splanchnic vein thrombosis, deep vein thrombosis, pulmonary embolism, and arterial thrombosis have also been seen [15]. The Pfizer COVID-19 vaccine, however, is an mRNA vaccine, and is therefore likely to be unrelated to the post-vaccine thrombotic reaction seen with other vaccines.

Outcome and Follow-up

The patient was placed on goal-directed medical therapy including intravenous furosemide 60mg three times daily, spironolactone 25mg daily, metoprolol 25mg daily, and sacubitril-valsartan 24-26mg twice daily for heart failure management. Intravenous dexamethasone 10mg daily was initiated along with oral hydroxychloroquine 200mg daily for concomitant SLE. Intravenous heparin was given for the LV thrombus. This was dosed based on daily Anti-Factor Xa activity levels rather than aPTT, which is falsely elevated in antiphospholipid syndrome. On discharge, intravenous dexamethasone was switched to oral prednisone 40mg daily, and the patient remained on hydroxychloroquine. The patient was placed on enoxaparin upon discharge and bridged to warfarin with a goal INR of 3-3.5. Lastly, the patient was placed on levetiracetam 750mg twice daily for seizure management.

The patient showed improvement with anticoagulation and corticosteroid treatment. Prior to hospital discharge, repeat TTE performed 3 weeks after the initial admission showed partial recovery of LVEF at 50%, improvement in mitral regurgitation, and no evidence of left ventricular thrombus. Repeat brain MRI showed stability of the hyperintensities seen prior. The patient was deemed stable for discharge and given recommendations to follow up with primary care, Neurology, Cardiology, Hematology, and Rheumatology. The patient was readmitted to an outside hospital 2 weeks after discharge with heart failure exacerbation and worsening seizures. She was evaluated with video EEG and found to meet the criteria for psychogenic non-epileptic seizures, though concomitant seizures could not be ruled out. The patient was discharged from that hospital in stable condition and has not had any further admissions.

## Discussion

Antiphospholipid syndrome may be a primary diagnosis (primary antiphospholipid syndrome) or may be seen with another systemic autoimmune disease, most commonly SLE (secondary antiphospholipid syndrome). This patient was diagnosed with concomitant SLE, and therefore was considered to have secondary antiphospholipid syndrome. Although the risk of thombotic events and pregnancy complications are similar between patients with primary and secondary antiphospholipid syndrome, patients with secondary antiphospholipid syndrome are more likely to have heart valve abnormalities, thrombocytopenia, and hypocomplementemia [16,17].

Antiphospholipid syndrome is associated with microvascular thrombosis, which can lead to widespread myocardial ischemia and dysfunction, similar to what was seen in this patient [5]. There have been a few autopsy reports of diffuse microthrombosis of the small intracardiac arterioles leading to cardiac necrosis in patients with antiphospholipid syndrome, both primary and secondary to SLE [18-20].

There are also several central nervous system manifestations of antiphospholipid syndrome, including stroke and transient ischemic attack due to vascular thrombosis or embolism [21]. Additionally, epilepsy may be associated with the presence of antiphospholipid antibodies. Although the primary mechanism by which this occurs is likely ischemic insult to brain parenchyma due to hypercoagulability, direct depolarization of neurons by antiphospholipid antibodies may also play a role [14].

Currently, anticoagulation is the primary focus in treatment of antiphospholipid syndrome and prevention of thrombotic events. Warfarin is dosed following a goal INR of 2.0-4.0, depending on the healthcare center [22]. However, due to the rare presentation of diffuse cardiomyopathy in antiphospholipid syndrome, there are no guidelines or recommendations for treatment or prevention of this manifestation [23]. Similarly, there is a lack of randomized controlled trials providing evidence about the treatment of central nervous system manifestations of antiphospholipid syndrome [21]. Therefore, we chose to initiate anticoagulation as described previously. Due to the concomitant SLE, this patient was also started on high-dose corticosteroid therapy and hydroxychloroquine.

While our patient did not meet the full criteria for CAPS, her presentation supports severe coagulopathy in the setting of known antiphospholipid syndrome, with a suspected immunologic trigger from recent COVID-19 immunization. There have been some case reports of CAPS after COVID-19 vaccination [24,25]. Though more research is needed to determine the immunogenicity of mRNA vaccines, it is plausible that the vaccine could have induced severe coagulopathy with concern for approaching CAPS.

## Conclusions

Antiphospholipid syndrome is characterized by vascular thrombosis and/or pregnancy morbidity. Patients with acute thrombotic episodes are generally treated with anticoagulation. However, some patients are found to have antiphospholipid syndrome on routine screening for other purposes, without any previous episodes of vascular thrombosis. Primary prevention is not recommended in this group. Catastrophic Antiphospholipid Syndrome (CAPS) is a severe disorder in which multi-organ small vessel thrombosis occurs over a short period of time. Treatment for CAPS includes anticoagulation, pulse-doses corticosteroids followed by a taper, and plasmapheresis and/or intravenous immunoglobulin. COVID-19 Vaccination may be an immunogenic trigger leading to CAPS in some patients.
